# The ‘captain of the men of death’, *Streptococcus pneumoniae*, fights oxidative stress outside the ‘city wall’

**DOI:** 10.1002/emmm.201303482

**Published:** 2013-11-04

**Authors:** Alexandra Gennaris, Jean-Francois Collet

**Affiliations:** Welbio and de Duve Institute, Université Catholique de LouvainBrussels, Belgium

**Keywords:** methionine sulfoxide, oxidative stress, *Streptococcus pneumoniae*, thioredoxin, virulence

We have come a long way since *Streptococcus pneumoniae* was first isolated in 1880. Over the years, the ability to combat this gram-positive bacterium branded ‘captain of the men of death’ by William Osler in 1918 has been greatly enhanced by the development of vaccines and antibiotics. However, despite the existence of these therapeutic strategies, *S. pneumoniae* continues to kill to this day. The bacterium, which is usually found as a resident of the human naso-oropharynx, causes indeed serious invasive diseases such as pneumonia, bacteraemia and meningitis, especially in young children, the elderly and immunocompromised individuals. Worldwide, it causes between 700,000 to 1 million child deaths every year (O'Brien et al, 2009). The limitations of existing vaccines, the emergence of antibiotic resistant clones, and the aging of the population will only make the problem worse, calling for new approaches against this pathogenic microorganism.

*S. pneumoniae* is an anaerobic bacterium. However, it is tolerant to the oxygen (O_2_) present in the respiratory tract and is equipped with antioxidant systems to fight reactive oxygen species (ROS). ROS, such as superoxide (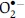) and hydrogen peroxide (H_2_O_2_), are produced by transfer of electrons to O_2_ following metabolic reactions or through the oxidative burst in neutrophils and macrophages. Like many other bacteria, *S. pneumoniae* possesses specialized antioxidant enzymatic squads that are in the front line to convert ROS to innocuous products unable to damage cellular components. The ROS detoxifying proteins that have been described in *S. pneumoniae* include a superoxide dismutase (SodA), a thiol peroxidase (TpxD) and an alkyl hydroperoxidase (AhpD). Interestingly, *S. pneumoniae* apparently lacks catalase, a ubiquitous H_2_O_2_ scavenger, and a response induced by oxidative stress (Yesilkaya et al, 2013). This is particularly intriguing given the fact that *S. pneumoniae* itself produces high amounts of H_2_O_2_, generated by the action of the pyruvate oxidase SpxB, to serve as a chemical weapon against the other bacteria present in the naso-oropharynx (Pericone et al, 2000).

ROS molecules can damage cells in many ways (Imlay, 2013). In proteins, the sulfur-containing amino acids cysteine and methionine are very susceptible to oxidation, which can lead to protein inactivation or degradation. Thus, *S. pneumoniae* possesses widely conserved repair systems to cope with oxidative damage inflicted to proteins. Thioredoxin (Trx), a ubiquitous oxidoreductase, plays a major role in these systems either by directly repairing oxidized cysteines (Collet and Messens, 2010) or by providing reducing equivalents to enzymes, known as methionine sulfoxide reductases (Msr), which reduce oxidized methionines (Ezraty et al, 2005). One Trx (TrxA) and one Msr (*Sp*MsrAB1) have been described in the cytoplasm of *S. pneumoniae* and partially characterized (Kim et al, 2009). Moreover, an additional Msr (*Sp*MsrAB2), anchored to the cytoplasmic membrane, has recently been identified and shown to be part of a pneumococcal repair system called CTM (short for CcdA, TlpA, MsrAB). This system also involves CcdA1, which belongs to a family of bacterial proteins transferring electrons across membranes, and a thioredoxin-like lipoprotein, TlpA (Andisi et al, 2012). Although the CTM system was shown to be important for resistance towards H_2_O_2_ and for virulence, its function remained unclear.

…Saleh et al (2013) add new pieces to the CTM puzzle and uncover a novel antioxidant artillery of *S. pneumoniae*.

**Figure 1:**
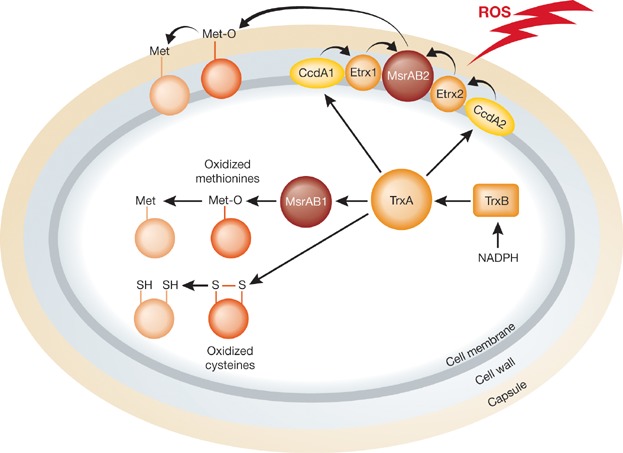
Like many other bacteria, *S. pneumoniae* possesses reducing enzymes such as thioredoxin (TrxA) to rescue oxidized proteins TrxA is maintained reduced by thioredoxin reductase (TrxB) at the expense of NADPH. In *S. pneumoniae*, TrxA donates electrons to the membrane proteins CcdA1 and CcdA2. The electrons are then transferred to Etrx1/Etrx2 and finally to MsrAB2, a surface-exposed methionine sulfoxide reductase. See the main text for more details.

In this issue of *EMBO Molecular Medicine*, Saleh et al (2013) add new pieces to the CTM puzzle and uncover a novel antioxidant artillery of *S. pneumoniae*. The first step of the study was the finding that the *S. pneumoniae* chromosome encodes an additional CcdA protein (CcdA2) and a TlpA homolog (Etrx2). For clarity, TlpA was renamed Etxr1. They found that Etrx1, Etrx2 and *Sp*MsrAB2 are exposed on the surface and that Etrx1 and Etrx2 are involved in two parallel pathways that provide reducing equivalents to *Sp*MsrAB2. Their results also suggest that electrons are donated to Etrx1 and Etrx2 by the membrane proteins CcdA1 and CcdA2 (Fig 1). Thus, electrons likely originating from the cytoplasm cross the membrane via CcdA1 and CcdA2, are transferred to Etrx1 and Etrx2 and are finally provided to the methionine regenerating enzyme *Sp*MsrAB2. Saleh et al went on to study the importance of the new reducing system in oxidative stress resistance and virulence using a set of mutants deleted for the various genes involved. They found that the absence of both pathways strikingly decreases the resistance of *S. pneumoniae* towards exogenously added H_2_0_2_. Moreover, mutants affected in both pathways have a dramatically reduced virulence in a mouse model of acute pneumonia and are significantly more phagocytosed by macrophages. Interestingly, impairment of either one of the Etrx1/Etrx2 pathways also has an impact, but clearly not to the extent observed when both pathways are affected. Thus, the authors bring to light a new extracellular reducing system composed of two complementary pathways that is essential for the virulence of *S. pneumoniae* and for its resistance to H_2_O_2_.

Accumulating evidence has shown that scavenging and repair systems are of extreme importance for bacteria to cope with their environment and with the immune response. Moreover, repair enzymes such as Trx and Msr have been demonstrated to play a role in the virulence of pathogens such as *Mycobacterium tuberculosis* and *Salmonella typhimurium* (Bjur et al, 2006; Denkel et al, 2011; Lee et al, 2009). However, most of the reducing systems for which a clear role in virulence has been demonstrated are present in the bacterial cytoplasm. In contrast, although extra-cytoplasmic antioxidant pathways have been described (Cho and Collet, 2013), it remained unclear whether they play an important role in bacterial virulence. Thus, the findings of Saleh et al bring a novel element to the antioxidant bacterial force by showing that an extra-cytoplasmic reducing system is essential for the virulence of a pathogen.

The paper also raises a number of interesting questions that require further investigation. For instance, one wonders if the Etrx proteins have other extracellular substrates than *Sp*MsrAB2. Also of particular interest would be the identification of the substrates of *Sp*MsrAB2 that need reduction of their oxidized methionines. Moreover, it would be judicious to further investigate the impact of extracytoplasmic reducing systems on the virulence of other major pathogens, these systems being the first line of defence towards the exogenous oxidative stress encountered during the immune response.

Not only the conserved Etrx1/Etrx2 system is an attractive target for the development of new antimicrobials, but it could also be useful for the development of new vaccines.

Finally, the study of Saleh et al provides a valuable contribution to the ongoing fight against *S. pneumoniae*. Not only the conserved Etrx1/Etrx2 system is an attractive target for the development of new antimicrobials, but it could also be useful for the development of new vaccines. Indeed, the existing vaccines, which are based on polysaccharide determinants of the pneumococcal capsule, cannot protect against all of the 90 capsular variants (serotypes) of the bacterium. Thus, targeting the surface-exposed Etrx1/Etrx2 system, which is common to all serotypes, could provide new means to claim victory over a dreaded human pathogen.

The authors declare that they have no conflict of interest.
